# Antioxidant and Cytotoxic Activities of Kudzu Roots and Soy Molasses against Pediatric Tumors and Phytochemical Analysis of Isoflavones Using HPLC-DAD-ESI-HRMS

**DOI:** 10.3390/plants11060741

**Published:** 2022-03-10

**Authors:** Saied A. Aboushanab, Vadim A. Shevyrin, Grigory P. Slesarev, Vsevolod V. Melekhin, Anna V. Shcheglova, Oleg G. Makeev, Elena G. Kovaleva, Ki Hyun Kim

**Affiliations:** 1 Institute of Chemical Engineering, Ural Federal University Named after the First President of Russia B. N. Yeltsin, Mira 19, 620002 Yekaterinburg, Russia; vadim.shevyrin@gmail.com (V.A.S.); grixa2000@yandex.ru (G.P.S.); e.g.kovaleva@urfu.ru (E.G.K.); 2 Innovative Center of Chemical and Pharmaceutical Technologies, Laboratory of Organic Synthesis, Ural Federal University Named after the First President of Russia B. N. Yeltsin, Mira 19, 620002 Yekaterinburg, Russia; melekhinvv@mail.ru (V.V.M.); ann257goldfinch@gmail.com (A.V.S.); 3 Department of Biology, Ural State Medical University, Repina 3, 620014 Yekaterinburg, Russia; larim@mail.ru; 4 Department of Gene and Cell Therapy, Institute for Medical Cell Technologies, Karla Marksa 22a, 620026 Yekaterinburg, Russia; 5 School of Pharmacy, Sungkyunkwan University, Suwon 16419, Korea

**Keywords:** isoflavones, kudzu roots, soy molasses, HPLC-ESI-HRMS, cytotoxicity, glioblastoma, osteosarcoma, rhabdomyosarcoma

## Abstract

Pediatric solid tumors (PSTs) are life-threatening and can lead to high morbidity and mortality rates in children. Developing novel remedies to treat these tumors, such as glioblastoma multiforme and sarcomas, such as osteosarcoma, and rhabdomyosarcoma, is challenging, despite immense attempts with chemotherapeutic or radiotherapeutic interventions. Soy (*Glycine max*) and kudzu roots (KR) (*Pueraria* spp.) are well-known phytoestrogenic botanical sources that contain high amounts of naturally occurring isoflavones. In the present study, we investigated the antioxidant and cytotoxic effects of the extracts of KR and soy molasses (SM) against PSTs. The green extraction of isoflavones from KR and SM was performed using natural deep eutectic solvents. The extracts were subsequently analyzed by high-performance liquid chromatography (HPLC)-diode array detector (DAD) coupled with high-resolution (HR) mass spectrometry (MS), which identified 10 isoflavones in KR extracts and 3 isoflavones in the SM extracts. Antioxidant and cytotoxic activities of KR and SM extracts were assessed against glioblastoma multiforme (A-172), osteosarcoma (HOS), and rhabdomyosarcoma (Rd) cancer cell lines. The KR and SM extracts showed satisfactory cytotoxic effects (IC_50_) against the cancer cell lines tested, particularly against Rd cancer cell lines, in a dose-dependent manner. Antioxidant activity was found to be significantly (*p* ≤ 0.05) higher in KR than in SM, which was consistent with the results of the cytotoxic activity observed with KR and SM extracts against glioblastoma and osteosarcoma cells. The total flavonoid content and antioxidant activities of the extracts were remarkably attributed to the isoflavone content in the KR and SM extracts. This study provides experimental evidence that HPLC-ESI-HRMS is a suitable analytical approach to identify isoflavones that exhibit potent antioxidant and anticancer potential against tumor cells, and that KR and SM, containing many isoflavones, can be a potential alternative for health care in the food and pharmaceutical industries.

## 1. Introduction

Cancer is considered the most crucial disease with rapidly increasing incidence worldwide, particularly among pediatric patients. Cancers affecting pediatric patients, including pediatric solid cancers (PSCs), are highly metastatic, demonstrate an inferior prognosis, and are largely resistant to current therapeutic regimens. Brain tumors, such as glioblastoma multiforme (GBM) and sarcomas, such as osteosarcoma (OS) and rhabdomyosarcoma (RMS), comprise approximately 40% of the pediatric solid tumors (PSTs) [1,2]. Despite immense efforts to develop various therapeutic interventions, progression to distant metastases is observed in approximately 50% of all patients [3,4,5]. Unfortunately, after metastasis, these patients are ineligible for surgical treatment, and their lifespan with or without treatment is less than 12 months [6].

Previous studies have acknowledged the use of radiotherapeutic and chemotherapeutic agents for the treatment of these tumors. Nevertheless, these therapeutic approaches are unfortunately toxic, ineffective due to resistance of tumor cells, besides being unsafe for human consumption [7,8]. Accordingly, there is an urgent need to discover innovative therapeutic strategies that can potentially suppress the growth of tumor cells with minor adverse effects. Medicinal plants, as an alternative therapy that is used in the food and pharmaceutical industries, are ultimately one of the best ways forward since they are associated with a low risk of cancer and cardiovascular complications [9,10]. Furthermore, some types of cancer develop and grow remarkably under the influence of endocrine hormones, such as estrogen, progesterone, or androgens. Therefore, hormone-based therapy is a potential treatment option for hormone-dependent cancers [11,12]. Phytoestrogens are secondary metabolites that initiate biological activities by mimicking the action of the human hormone, estrogen or 17-β-estradiol. Soy (*glycine max*) and kudzu roots (KR) (*Pueraria* spp.) are phytoestrogenic botanical sources that contain high amounts of naturally occurring polyphenols, namely isoflavones [13,14,15]. Accumulating evidence has substantially confirmed the fundamental health benefits of the consumption of these isoflavones. Owing to their various health-promoting properties, such as cancer prevention, reduction of oxidative stress, and alleviation of osteoporosis, isoflavones are considered potential targets in the pharmaceutical industry, which can be obtained via a nutritious diet [16,17]. Indispensably, soy molasses (SM) and parts of kudzu, e.g., KR, are regarded as waste in the manufacturing process; hence, they need to be recycled and repurposed [18].

The pharmaceutical potency of plant-derived compounds is remarkably dependent on their dose, chemical profile, and bioavailability [19]. Hence, it is essential to determine the unique profile and quantify the plant-derived isoflavones to assess therapeutic interventions in vitro and in vivo. Many studies have attempted to isolate and characterize isoflavones obtained from SM syrup and KR [20,21,22]. To accomplish this, appropriate extraction technology and sensitive analytical method are sought to identify the low concentrations of isoflavones in these plant preparations [23]. Recently, deep eutectic solvents as extraction solvents and ultrasonic assistance, a recently developed technology, have been in the limelight as favorable and effective extraction approaches. Alternatives involving the utilization of these green extraction materials, for example, choline chloride and citric acid, could assist in mitigating a variety of extraction conditions, thereby potentially minimizing the use of toxic solvents and lengthy extraction procedures [24]. Previous studies have attempted to analyze phytoestrogenic compounds using high-performance liquid chromatography (HPLC)-MS/MS systems [20,25]. Because of the difficulty to obtain satisfactory peaks using a single chromatographic methodology and the low sensitivity of other reported analytical techniques [23,26], HPLC-diode array detector (DAD), electrospray ionization (ESI), and high-resolution (HR) mass spectrometry (MS) in positive and negative ion modes were adopted to analyze the analytes of interest in both KR and SM extracts. Interestingly, HRMS is a more flexible tool than MS/MS system and can identify compounds with high confidence, besides detecting the unknown or unexpected compounds. In addition, compared to HPLC-ESI-MS, HPLC-ESI-HRMS can resolve the false positive findings that cannot always be avoided when adhering to established MS/MS techniques [27]. In this study, daidzein, genistein, puerarin, formononetin, and biochanin extracted from both plant preparations were tested using an HPLC-ESI-HRMS analytical instrument.

Recently, various studies have been conducted to survey the cytotoxic activity of plant-derived compounds, such as isoflavones, against human cancer cells [28,29]. Despite the extensive investigation of their promising biological potentials, the study of possible cytotoxic potency of isoflavones remains important. Within this context, due to the presence of polyphenols and natural flavonoids, plant-derived antioxidants exert a chemoprotective effect against oxidative damage induced by cancer invasion [30]. Although many herbal extracts, fractions, and isolated molecules aid in the accumulation of free radical scavengers, the therapeutic cytotoxic interventions exploiting isoflavones are still limited, particularly against PSCs. Several in vitro investigations have revealed the cytotoxic and apoptosis-inducing effects of isoflavones in different cancer cell lines. Despite attempts to characterize plant-derived isoflavones [31,32], the full chemical profile and antiproliferative cytotoxic effect of KR and SM against glioblastoma, osteosarcoma, and rhabdomyosarcoma tumor cells have not been explored. Based on the previous findings and gaps in the current state of knowledge, the objective of this study was to evaluate the fractions of isoflavones from KR and SM using green extraction and HPLC-ESI-HRMS-guided fractionation and investigate their cytotoxic efficiency against PSTs in vitro.

## 2. Materials and Methods 

### 2.1. Chemicals, Reagents, and Equipment

Reference standards of daidzein, genistein, puerarin, formononetin, and biochanin A with a purity of ≥98% were purchased from Sigma Aldrich (St. Louis, MO, USA) and used without further purification. Methanol (99.9%, for HPLC gradient), formic acid (98.0–100%, Puriss., for meeting analytical specifications of DAC and FCC), acetic acid (99.8%, for HPLC), and acetonitrile (99.9%, HPLC) were purchased from Sigma Aldrich. Choline chloride (99%; pharmaceutical grade) was purchased from Acros Organics, Geel, Belgium. Citric acid (99%, food-grade) was purchased from Sigma Aldrich. Quercetin, gallic acid, and 2, 2-diphenyl-1-picrylhydrazyl (DPPH) were purchased from Sigma-Aldrich. Ethyl acetate and ethanol were purchased from Himreaktivsnab company, Ufa, Russia. All other reagents and chemicals used in this study were of analytical grade. An ultrasonic cleaner and laboratory centrifuge Elma PE-6926 with a 10 × 5 mL rotor were used for the extraction process. A spectrophotometer Shimadzu-UV 1800 (Chiyoda-ku, Tokyo, Japan) was used as an analytical tool for the evaluation of total polyphenol content and antioxidant activity. A hot oven (Dry Oven UN55, Memmert, Schwabach, Germany) was used to dry the samples. In addition, HPLC-ESI-HRMS was used to quantify isoflavone content.

### 2.2. Plant Materials

A mixture of *Pueraria lobata* and *Pueraria mirifica* or dried KR was purchased from Xi’an Sgonek Biological Technology (Xian, Shanxi, China). Similarly, SM syrup (*Glycine max*), a by-product of soy protein concentrate, was supplied by Agroproduct CJSC, Kaliningrad, Russia, which was stored at −20 °C until further use.

### 2.3. Preparation of Natural Deep Eutectic Solvents (NADESs)

The NADES-1 solution used to extract isoflavones from KR comprised a two-component mixture of choline chloride and citric acid at a 1:2 molar ratio. For the extraction of isoflavones from SM, a natural deep eutectic solvent 2 (NADES-2) mixture was prepared by mixing equimolar quantities of choline chloride and citric acid.

Briefly, the two-component mixtures of NADES-1-and 2 were transferred into a glass seal and distilled water (20% and 30%, respectively) was added. The final mixtures were heated at 60–80 °C under constant stirring until transparent solutions were obtained. The prepared NADESs were stored in the dark until further use [33].

### 2.4. NADES-Based Ultrasound-Assisted Extraction of KR and SM

The NADES-based ultrasound-assisted extraction procedure was used for the extraction of isoflavones from KR and SM, as described by Dai et al. with minor modifications [34,35]. Approximately 1 g of KR and SM was accurately weighed into a 50 mL beaker, to which 20 mL and 30 mL of NADES solution were immediately added, respectively. The mixtures were processed by ultrasonic extraction at a frequency of 37 kHz and power of 580 W at 60 °C for 3 h using ultrasonic extraction equipment. Isoflavones were gradually extracted into the NADES phase to obtain a viscous suspension. The suspension was centrifuged at 6000 rpm for 10 min to separate the solid and liquid phases. NADES supernatants were washed using ethyl acetate thrice in a 1:3 (*v/v*) ratio in a separating funnel and resultant upper layers were concentrated with a rotatory evaporator until complete dryness. The final extract was stored at −20 °C until further use.

A certain amount of the resulting dry powder was diluted in methanol. The concentrations of the isoflavones in the final solution were determined by HPLC using calibration curves and HRMS. The same procedure was performed in triplicates for all experimental treatments.

Extraction yields (E_y_) were calculated as follows [36]:

(1) $$\mathrm{Ey}=\frac{C_{f}\times V_{s}}{m_{s}}$$

where C_f_ refers to the concentration of isoflavones found in the NADES fractions using HPLC analysis, V_s_ refers to the diluted suspension volume, and m_s_ refers to the mass of the test sample.

### 2.5. Quantitative Determination of Isoflavones Using HPLC-DAD Method

Quantification of isoflavone content in the extracts was performed using an HPLC Agilent 1260 Infinity II system consisting of a quaternary pump (Model G7111B), diode array ultraviolet (UV) detector (Model G7117C) coupled with DAD analysis software, an autosampler (Model G7129A) with an integrated column compartment, and vacuum degasser module. A Poroshell 120 EC-C18 (3.0 mm × 100 mm, 2.7 μm, Agilent Technologies, p/n 695975-302 (Santa Clara, CA, USA) reversed stationary phase column with an additional 5-mm guard column was used for isoflavone separation. The mobile phase consisted of solvent A, containing 0.1 % (*v/v*) acetic acid in the water, and solvent B, containing 0.1 % acetic acid (*v/v*) in methanol. Linear gradient elution of solvent B was applied from 5% up to 100 % over 20 min, which was maintained for 1.5 min at a flow rate of 0.7 mL/min. The temperature of the column was maintained at 30 °C, and the injection volume was 5 μL. Isoflavones were detected at a wavelength of 254 nm. The chromatographic peaks of isoflavones in the extracts were identified based on the retention intervals and UV spectra of the peaks corresponding to the reference standards of daidzein, genistein, puerarin, formononetin, and biochanin A, with a concentration range of approximately 0.04–1.6 μg/mL. The calibration curves showed linear dependence in the indicated concentration range (R^2^ not less than 0.999). A quantitative analysis of the five peaks that were remarkably characteristic of isoflavone content in the extracts was performed. The isoflavone yield was expressed in g/100 g of KR or SM for the ethyl acetate fractions of both extracts.

### 2.6. Analysis of Isoflavones Using HPLC-ESI-HRMS Method

Chemical identification of the bioactive compounds in both extracts was performed by HPLC-ESI-HRMS and MS/MS analysis using an Agilent 1290 Infinity II HPLC system connected with a quadrupole time-of-flight (Q-TOF) accurate mass detector (Agilent 6545 Q-TOF LC-MS, Agilent Technologies, Santa Clara, CA, USA). The HPLC conditions were established as described above, except for the column and the flow rate. Chromatographic separation was performed using a Poroshell 120 EC-C18 (2.1 mm × 100 mm, 2.7 μm, Agilent Technologies, p/n 695775-902, Santa Clara, CA, USA) reversed stationary phase column with an additional 5 mm guard column at a flow rate of 0.4 mL/min. The injection volume was 1 μL. Q-TOF instrument was operated with an ESI source in positive and negative ion modes using the following conditions: drying gas temperature, 350 °C (nitrogen, 10 L/min); nebulizer pressure, 40 psi; capillary voltage, 3500 V; and fragmenter voltage, 90 V. In the MS/MS mode, the quadrupole was adjusted to isolate precursor ions with a bandwidth of Δ *m/z* = 1.3. The CID spectra of the precursor ions were recorded with collision energy (CE) in the range of 20–60 eV. The collision cell was filled with nitrogen (99.999%). Ions were scanned in the mass range of 100–1000 Da in the MS mode and 40–700 Da in the MS/MS mode. The TOF detector was operated in EDR (2 GHz) mode, and the acquisition was 1.5 spectra/s. The mass spectrometer was adjusted, and the mass measurement accuracy was corrected automatically in accordance with the instruction manual of the device and using recommended standard solutions (Agilent, part. numbers G1969-85000 and G1969-85001).

### 2.7. DPPH Radical Scavenging Activity (Spectrophotometry and Electron Spin Resonance Spectroscopy)

The DPPH radical scavenging effect was estimated as previously described by Chen et al. (2013) with minor modifications [37]. DPPH solution (0.5 mM) was prepared in methanol and 200 μL of the DPPH solution was added to 25 μL of each test sample. The mixtures were allowed to react at ambient temperature, and absorbance was immediately measured using an EPR spectrometer (EPR Elexys E-500) (Bruker Biospin, Karlsruhe, Germany). Ascorbic acid, a well-known antioxidant, was used as the positive control in the assay. The antioxidant activity was calculated by plotting a standard curve and was determined in mM ascorbic acid equivalency. Three different concentrations of ascorbic acid solutions (1, 0.5, and 0.25 mM) and methanol were used as the standards and blank, respectively. The intensity of the EPR spectrum decreased with an increase in the concentration of ascorbic acid, which, in turn, increased the percentage inhibition (I, %). The percentage of inhibition of the EPR spectrum was calculated using the following equation:

(2) $$I\;\left(\%\right)=\frac{I_{0}-I}{I_{0}}\times 100$$

where I_0_ refers to the double integral of the EPR signal of the water-methanol mixture radical (225 µL of DPPH in methanol), and I refers to the double integral of the EPR signal of a mixture of 200 µL of DPPH in ethanol and 25 µL of the sample. The EPR spectrum of DPPH changed over time in the process of reaction with an antioxidant and was recorded over five minutes.

Similarly, after incubating the extract mixtures for 90 min in dark, the DPPH radical reaction was monitored by recording the absorbance at 514 nm using spectrophotometric analysis [38]. The decreased absorbance of the reaction mixture indicates an increased percentage of free radical scavenging activity. The percentage of inhibition or free radical scavenging activity was calculated using the following formula:

(3) $$\mathrm{Inhibition}\;\left(\%\right)=\frac{\mathrm{control}\;\mathrm{absorbance}\;-\;\mathrm{sample}\;\mathrm{absorbance}}{\mathrm{control}\;\mathrm{absorbance}}\times 100$$

The control comprised only methanol and DPPH solutions. The percentage of inhibition was calculated from the graph of the inhibition curves. All reactions were monitored in duplicate, and the values are expressed as the mean ± standard deviation (S.D.).

### 2.8. Total Polyphenol Content

The total phenolic content of KR and SM extracts was determined according to a previously described method with minor modifications [39]. Briefly, sample (0.25 mL) or standard gallic acid (0, 50, 100, 150, 250, and 500 mg/L) solutions were pipetted into assay tubes. Folin Ciocalteu solution (0.5 mL) and distilled water (5.5 mL) were then mixed and homogenized. A total of 1 mL of Na_2_CO_3_ (20%) was added after 5 min of incubation. Assay tubes were incubated at 20 °C for 2 h, and absorbance was measured at 765 nm within 30 min against a blank (distilled water) using a spectrophotometer (UV-1800, Shimadzu Chiyoda-ku, Tokyo, Japan). The total phenol content was calculated from the standard curve of gallic acid (y = 0.0038x + 0.0487, R^2^ = 0.9982), and the results were expressed as mmol/L of gallic acid equivalents per gram of the extract.

### 2.9. Total Flavonoid Content (NaNO_2_–Al (NO_3_)_3_–NaOH Colorimetric Method)

The total flavonoid content was evaluated as described by Huang et al., with minor modifications [40]. Briefly, 0.5 mL of extract was mixed with 2 mL of 30% ethanol and 0.15 mL of NaNO_2_ (5%, *w/v*). After 5 min of reaction, the mixture was reacted with 0.15 mL of Al(NO_3_)_3_ (10%, *w/v*) for 6 min. Then, 2 mL of 1M NaOH was added, and the mixture was adjusted to 5 mL with 0.2 mL of 30% ethanol. After incubation at room temperature for 10 min, the absorbance was measured at 510 nm. Al(NO_3_)_3_ and NaOH solutions were substituted with the same amount of 30% ethanol in the blank. The total flavonoid content of the samples was expressed as quercetin equivalent (mmol/L) (y = 0.0007x + 0.0086, R^2^ = 0.9954), and the calibration curve ranged from 0 to 500 μg/mL.

### 2.10. Assessment of Biological Activity

#### 2.10.1. Cell Lines and Culturing

Experiments were performed with cultured cells of human glioblastoma (A-172, ATCC CRL-1620) [41,42], human osteosarcoma (Hos, ATCC CRL-1543) [43] and human embryonic rhabdomyosarcoma (Rd, ATCC CRL-136) [44] obtained from the Institute of Cytology RAS, Russia. The cells were cultured in a DMEM/F-12 medium containing 10% fetal bovine serum at 37 °C, 5% CO_2_, and 98% humidity. Subculturing using 0.25% trypsin solution was performed when the culture reached ≥90% confluency. Both extracts were dissolved in DMSO and diluted with DMEM/F-12 culture medium and 10% fetal bovine serum to obtain the following concentrations: 16, 32, 64, 128, 256, 512, 1024, and 2048 μg/mL. The process used to obtain the SM extract allowed us to investigate a concentration range of up to 1024 μg/mL. In all cases, the DMSO concentration in the final solution did not exceed 1%.

#### 2.10.2. Cell Viability Assessment

Tumor cells were pre-seeded in 96-well plates at a seeding concentration of 4 × 10^3^ cells/well. After 24 h, the extracts were added to the wells of the plate at a predetermined concentration range. Then, the cells were incubated for 72 h, after which a solution of MTT (3- (4,5-Dimethyl-2-thiazolyl)-2,5-diphenyl-2H-tetrazolium bromide) 20 μL (5 mg/mL) was added to the cultures per hole. After 2.5 h, the medium was removed from the wells, and 200 µL of a 1:1 mixture of DMSO and isopropanol was added. The optical density (OD) was measured using a microplate spectrophotometer (Thermo Fisher Scientific, Waltham, MA, USA) at a wavelength of 570 nm.

The percentage of viable cells was calculated as follows [45]:

(4) $$\%\;\mathrm{of}\;\mathrm{viable}\;\mathrm{cells}=\frac{\mathrm{OD}\;\mathrm{of}\;\mathrm{sample}-\mathrm{OD}\;\mathrm{of}\;\mathrm{blank}}{\mathrm{OD}\;\mathrm{of}\;\mathrm{intact}\;\mathrm{cell}-\mathrm{OD}\;\mathrm{of}\;\mathrm{blank}}\times 100$$

### 2.11. Statistical Analysis

All extractions were performed twice, and all measurements were performed in triplicates. Values are presented as the mean ± standard deviation. All the parameters were analyzed at a 95% significance level (*p* < 0.05) using GraphPad Prism 08.0.2 software (San Diego, CA, USA). Statistical analysis of cytotoxicity was performed using package R (version 4.0.3) and the RStudio program (version 1.4.1106 © 2009–2021 RStudio, PBC). The cytotoxicity index (IC_50_) was calculated by plotting dose-effect curves using the “drc” package [46].

## 3. Results and Discussion

### 3.1. Extraction, Recovery, and Quantification of Isoflavones

Phytoestrogens are naturally found in bioactive compounds with diverse biological functions. Isoflavones are phytoestrogenic compounds of considerable interest because of their ability to selectively act as estrogen agonists or antagonists. *Pueraria* roots and *Glycine max* are botanical phytoestrogenic sources well-known for their antioxidant and antitumor potentials. The estrogenic effect of these phytoestrogens has been attributed to their isoflavone-rich content. Hence, it is fundamentally important to recover these bioactive ingredients to introduce novel pharmaceutical preparations that show potent antioxidant activities and reduce the risk of disease complications.

**Figure 1:**
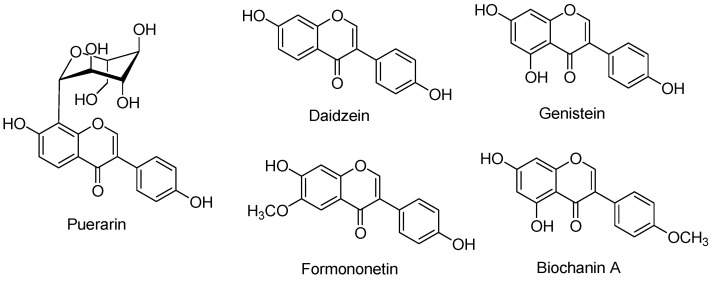
Chemical structures of isoflavones, puerarin, daidzein, genistein, formononetin, and biochanin A.

In the present study, we aimed to recover isoflavones from KR and SM and fractionate their extracts, rather than optimize the extraction conditions. The botanical sources (KR and SM) were characterized by the presence of five main isoflavones (puerarin, daidzein, genistein, formononetin, and biochanin A) (Figure 1). These bioactive components, particularly puerarin, daidzein, and genistein, have been developed as pharmaceutical interventions in previous studies. One-pot green extraction method demonstrated efficient extraction of isoflavones using natural solvents compared to that observed with the use of conventional solvents. Ethyl acetate was used for the recovery of isoflavones based on previously published studies, which showed that ethyl acetate contained the highest quantity of phenols and flavonoids [28]. In addition, HPLC-DAD is a reliable method for identifying and quantifying major compounds based on the corresponding internal standards. Similarly, HPLC-DAD is a suitable methodology for quantifying phytoestrogens in different samples.

### 3.2. Quantification of Isoflavones in KR and SM Extracts Using HPLC-DAD

To quantify isoflavones in the extracts, a calibration curve was plotted for five different isoflavones. The experimental data showed a coefficient of determination (R^2^) value of more than 0.9996 for all standards, as shown in Table 1. This indicates that this model was optimized and applicable for describing the response of the experiment to five isoflavones. Representative HPLC-UV chromatographic profiles of standard samples, including puerarin (A), daidzein (B), genistein (C), formononetin (D), and biochanin A (E), were plotted for analysis (data not shown).

**Figure 2:**
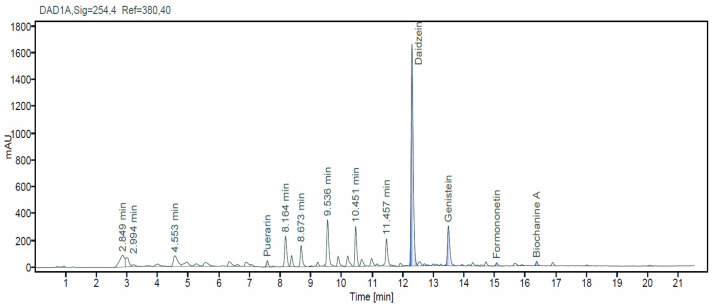
Representative HPLC-UV chromatographic profile for NADES extracts of kudzu roots.

NADES extracts of KR were analyzed using the HPLC-DAD system, as shown in Figure 2. The five main isoflavones were efficiently separated by HPLC and identified as daidzein, genistein, puerarin, formononetin, and biochanin A, according to the retention intervals of the respective isoflavone standards. The components identified in the extract after ethyl acetate fractionation were daidzein, genistein, puerarin, and formononetin. The determined contents were consistent with those observed by Zhang et al. [47]. KR accumulates large amounts of isoflavones (~1.8–12%), including puerarin and daidzein. The total concentration of isoflavones in KR was recorded at 1.09 ± 0.006%. One-pot green extraction of KR showed that the concentration of isoflavones was higher than that obtained by Tong-Rong using conventional extraction methodology (0.33%) [37]. However, Zhang et al. reported a higher concentration of isoflavones in KR (1.86%) using ethanol as an extractant [47].

**Figure 3:**
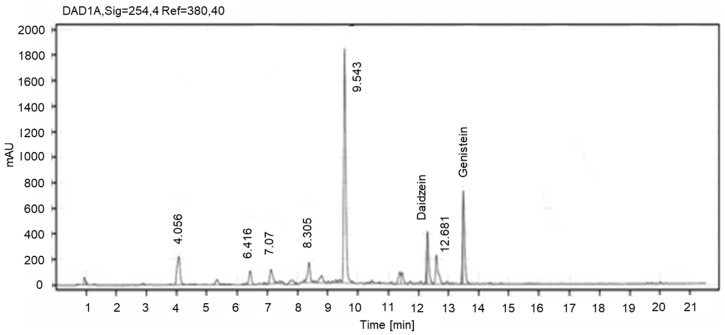
Representative HPLC-UV chromatographic profile for NADES extracts of soy molasses.

NADES extracts of SM were similarly analyzed using the HPLC-DAD method. Daidzein and genistein were identified among the five standards in SM extracts, as shown in Figure 3 [23]. The concentration of daidzein and genistein in SM extracts was also quantified as 0.67%. This value was significantly higher than that reported by Gu et al. [48]. The isoflavones identified in the ethyl acetate fractions of the KR and SM extracts were also quantified (Table 2). Importantly, both one-pot green extraction and fractionation using ethyl acetate improved the yield of isoflavones from both KR and SM compared to that observed in previous studies [34,45].

### 3.3. HPLC-ESI-HRMS Analysis

The HPLC-ESI-HRMS was established and validated for the evaluation of different isoflavones (daidzein, genistein, puerarin, formononetin, and biochanin A) [23]. This methodology is efficient because of the satisfactory chromatographic determination of a wide variety of bioactive ingredients with high estrogenic activity. This analytical approach is ultimately flexible and suitable for isoflavone quantification and could be practically utilized to estimate the association between isoflavone administration and various biological activities. Moreover, this method, when performed in positive ion mode, is crucial for detecting 64 different polyphenolic compounds [49].

According to the results of the HPLC-ESI-HRMS analysis, 10 compounds were identified in the extract derived from KR. The obtained data are summarized in Table 3. Based on the areas of chromatographic peaks, among the compounds identified using standard samples, daidzein and genistein were the main isoflavones found in the studied extract derived from KR. Formononetin and puerarin were present in lower quantities. The content of the remaining detected compounds was also relatively low. Daidzein and genistein were also the main isoflavones found in the SM extract (Table 4).

In the current study, KR and SM extracts were analyzed using the HPLC-ESI-HRMS method to obtain more extensive data on the composition of the extracts compared to those obtained using HPLC-DAD. Compounds with a peak response higher than 5 × 10^3^ counts and their calculated gross formula corresponding to the elemental composition of C_x_H_y_O_z_ with an error of no more than 5 ppm for the *m/z* value of the major isotope were considered in the current experimental analysis. The compounds in the extracts were identified based on the measured values of the accurate masses of the precursor ions and characteristic product ions in the CID spectra (error limit of ±5 ppm), including a detailed interpretation of the spectra using literature sources [50,51] and also by comparing with the spectra of standard samples and the spectra derived from the Metlin AM PCDL database (for Agilent) [52]. Additionally, the acquired MS/MS data were converted to the mzXML format and submitted to the Global Natural Products Social Molecular Network (GNPS) [53]; http://gnps.ucsd.edu, accessed on 8 December 2021, an online system for the identification of connections using open-access spectral databases.

In the HPLC-ESI-HRMS analysis, biochanin A was not found in the extract of KR; however, the chromatogram showed a peak [M − H]^−^ with *m/z* 283.0612 (C_16_H_12_O_5_) corresponding to an isomer of biochanin A with less retention time relative to the standard biochanin A. In the CID spectrum (Figure S1) of the detected compound, a methyl radical elimination characteristic of methoxylated isoflavones aided in the formation of the radical anion [M − H − CH_3_]^−●^, which further decayed with a sequential release of H^●^, CO, and CO_2_ [54,55] forming ions similar to those observed in the spectrum of biochanin A. At the same time, the ions formed as a result of retro-Diels-Alder (RDA) reactions [^0.3^B − CH_3_] ^−●^ with *m/z* 148.0166, [^1.3^A]^−^ with *m/z* 135.0088, and [^0.3^A]^−^ with *m/z* 119.0139 (Figure S2) indicated the presence of only one hydroxy group in ring A and one hydroxy and methoxy group in ring B according to the accepted nomenclature [50,54]. This compound may be a hydroxylated B-ring derivative of formononetin; however, the determination of the exact structure of the compound was difficult without the isolation of individual compounds.

A methylated isoflavone with a semi-formal C_16_H_12_O_6_ was also identified by GNPS spectral libraries [53], namely tectorigenin (Figure S3). The CID spectrum (Figure S3) of the protonated molecule was consistent with the library data. The spectrum showed an intense signal corresponding to [M + H − CH_3_]^+●^, whose decay proceeded with the release of H^●^ and neutral losses of H_2_O and CO, as well as ion signals [^1.3^A − CH_3_]^+●^ with *m/z* 168.0053, [^1.3^B]^+●^ with *m/z* 118.0413, and [^1.4^A − CH_3_]^+●^ with *m/z* 140.0104, which were distinctly characteristic of tectorigenin.

The CID spectra of the deprotonated molecule of the compound with the empirical formula C_17_H_14_O_5_ (Figure S4) showed a sequential release of two methyl radicals, leading to the appearance of intense ion signals [M − H − CH_3_]^−●^ with *m/z* 282.0534 and [M − H − 2CH_3_]^−^ with *m/z* 267.0299. The decay of the latter was characterized by consecutive losses of CO and CO_2_ molecules. The fragmentation pattern of the compound was identical to that observed in the spectra of 7-methyl ether of retusin and its isomer 7-hydroxy-8,4′-dimethoxyisoflavone presented in the GNPS spectral libraries [53] and Metlin AM PCDL [52]. The detected compound was likely a demethylated isoflavone with a similar structure, but the mutual arrangement of the substituents cannot be realized without the isolation of individual compounds. Along with the signal of genistein, the chromatogram of the KR extract revealed a minor peak corresponding to its isomeric compounds with less retention time (*t*_R_ = 9.6 min), whose spectra were identical to those of genistein.

In the KR extract, genistein was detected not only in the free form but also in two glycosidic forms with the same gross formula C_21_H_20_O_10_ (MW 432 Da) (Table 3). The CID spectrum (Figure S7) of the deprotonated molecule of the compound with a retention time *t*_R_ = 7.9 min ([M − H]^−^ = 431.0984) obtained at low collision energy (20 eV) demonstrated an intense signal of the Y_o_ ion^−^ with *m/z* 269.0455, corresponding to the loss of glycan residues C_6_H_10_O_5_ (162 Da). This finding confirmed the attachment of hexose to aglycone in the hydroxyl group [50,56,57]. The [Y_o_ − H]^−●^ ion formed as a result of the homolytic bond break in C–O ion [Y_o_ − H]^−●^ with *m/z* 268.0377 showed a high-intensity signal in the spectrum, which allowed us to hypothesize the structure of 7-O-glycoside [50,56,57].

In addition, the spectrum showed signals corresponding to ions formed as a result of bond breaking in the glycosidic part of the molecule: ^0.2^ X^−^ with *m/z* 311.0561 ([M − H − C_4_H_8_O_4_]^−^) and ^0.1^X^−^ + 2H with *m/z* 283.0612 ([M − H^−^ C_5_H_8_O_5_]^−^) [57]. When the collision energy increased, the relative intensity of the radical anion signal [Y_o_ − H]^−●^ increased, and simultaneously, [Y_o_ − 2H]^−^ ion signals appeared in the spectrum with *m/z* 267.0299 (Figure S8). Fragment ions with *m/z* values such as 240.0428, 239.0350, 224.0479, 223.0401, and 211.0401 formed as a result of the sequential elimination of CO and CO_2_ from the corresponding aglycon ions y_o_- and [Y_o_ − H]^− ●^ were also observed in the spectrum. The spectrum demonstrated diagnostic ions for genistein [^0.3^ B − H]^−●^ with *m/z* 132.0217 and ^0,3^ B^−^ with *m/z* 133.0295, formed by cleavage of the C ring as a result of the RDA reaction [57].

The CID spectra (Figures S9–S11) of the protonated molecule of this compound ([M + H] ^+^ = 433.1129) showed the signal of the Y_o_^+^ion with *m/z* 271.0601, which was formed as a result of the loss of hexose C_6_H_10_O_5_ (162 Da). The set of signals of fragmentary ions with lower values of *m/z* recorded in the spectra at higher values of collision energy (40–60 eV) was in line with the CID spectrum of genistein, as well as the spectrum of genistein-7-*O*-glucoside observed in a previous study [57]. Thus, the detected compound C_21_H_20_O_10_ (*t*_R_ = 7.9 min) was identified as genistein-7-O-glucoside.

For an isomeric compound with a *t*_R_ of 7.1 min, no intense signals corresponding to the ions of aglycon Y_o_^+^, Y_o_^−^, and [Y_o_ − H]^−●^ were observed in the CID spectra (Figures S12 and S13). This suggests the presence of a C-glycoside structure, in which the carbohydrate residue was bound to the aglycone through the C-C bond. At the same time, in the CID spectra (Figures S14–S18), the protonated molecule of the compound showed characteristic ions owing to the fragmentation of the glycosidic part of the molecule [50]. First, the elimination of water molecules with the formation of intense ions [M + H − H_2_O]^+^ with *m/z* 415.1021, [M + H − 2 H_2_O]^+^ with *m/z* 397.0918, and [M + H − 3H_2_O]^+^ with *m/z* 379.0818 should be noted. Based on the high intensity of ion signals [M + H − H_2_O]^+^ and [M + H − 2H_2_O]^+^, compared to the signal precursor ion in the spectrum (Figure S14) observed at low collision energy (20 eV), it is possible to speculate the probable binding of the carbohydrate with aglycone at position 8 of the latter (8-*C)* [58,59]. Similarly, the spectra showed signals of ^0.2^X^+^ ([M + H − 120]^+^) with *m/z* 313.0707 and ^0,1^X^+^ ([M + H − 150]^+^) with *m/z* 283.0601, as well as other characteristic ions formed as a result of the rupture of the bonds of the hexose ring [58,59]. The CID spectra of the compound obtained in the negative ion mode (Figures S12 and S13) demonstrated intense ion signals of ^0.2^X^−^ ([M – H − 120]^−^) with *m/z* 311.0561 and [^0.2^X^−^ − CO]^−^ with *m/z* 283.0612, which confirmed the hypothesis of the structure of the compound. The spectrum obtained at CE 40 eV (Figure S13) contained an ion ^0,3^B^−^, a diagnostic ion for genistein with *m/z* = 133.0295, and also showed a high similarity (cosine 0.90) with the spectrum genistein-8-*C*-glucoside published in GNPS spectral libraries [60].

The main components of the SM extract were genistein and daidzein, which were identified by comparing their CID spectra with those of the standard solutions, as well as genistein-7-O-glucoside, for which a high content in the extract was expected (Table 4). The CID spectra of genistein-7-*O*-glucoside showed that SM was identical to KR extract (Figures S7–S11).

To the authors’ knowledge, to date, there have been only two studies on identifying the analytical compounds in *Pueraria* species using HRMS methods. For instance, HPLC-HRMS with solid-phase extraction and nuclear magnetic resonance spectroscopy (HPLC-HRMS-SPE-NMR) have been proven successful to identify 21 known compounds and two new compounds in kudzu roots [61]. However, these KR have been conventionally extracted with methanol as a solvent without further purification, consequently resulting in obtaining a different chromatographic profile. Similarly, ultra-performance liquid chromatography-quadrupole-orbitrap high-resolution mass spectrometry (UPLC-Q-Orbitrap HRMS) method was recently employed to screen out 16 compounds including 4 of them beyond the established standard library [62]. On the other side, no reported studies have been reported about the analysis of SM extract using HPLC-ESI-HRMS. Nevertheless, a non-purified filtered soy molasses was previously subjected to HPLC-ESI_MS analysis and resulted in identifying 15 compounds including novel malonyl isoflavone glycosides [48]. To sum up, the chemical chromatographic profiles of KR and SM could be highly dependent on extraction technique, source of substrates, purity of extracts, and method of analysis.

### 3.4. DPPH Radical Scavenging Activity

**Figure 4:**
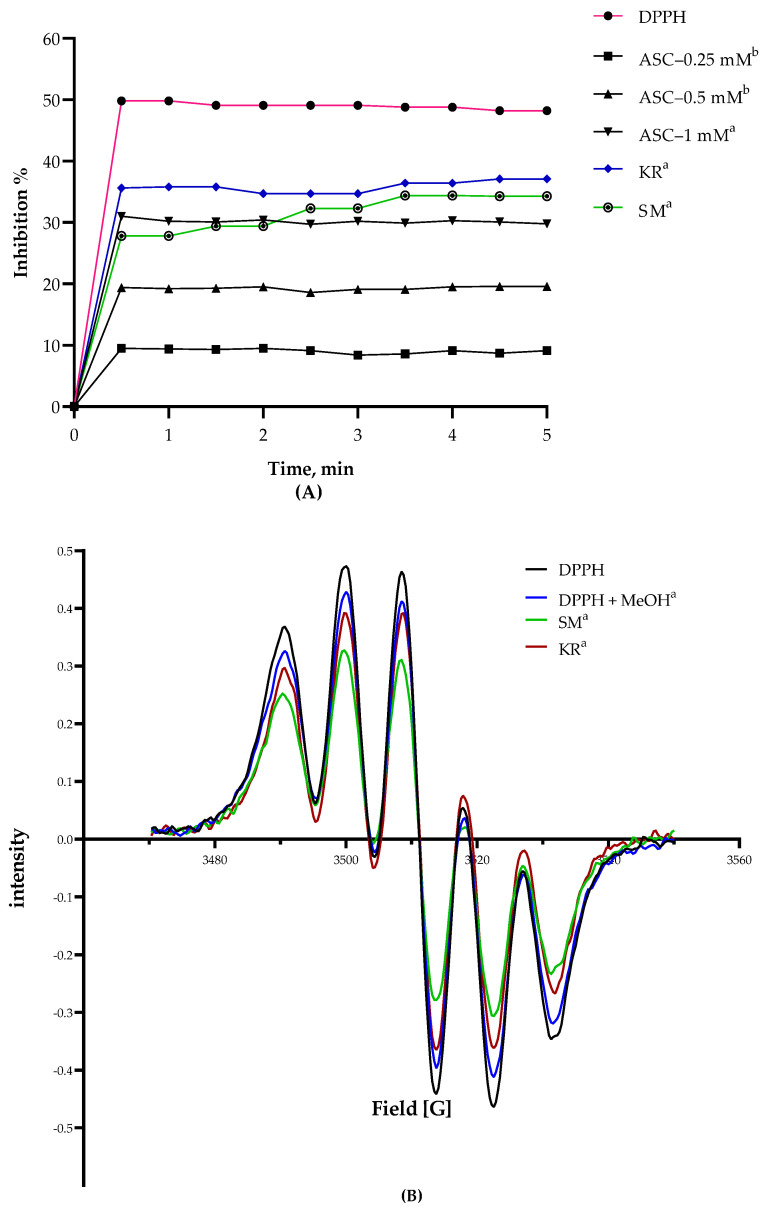
Inhibition percentage of kudzu roots (KR) and soy molasses (SM) extracts compared to standard ascorbic acid (ASC). DPPH for quantifying the intensity and ascorbic acid equivalency (**A**), and EPR spectra (**B**) of DPPH in different environments. Data are shown as mean ± SD. ^a,b^ Means that do not share the same letter in each column are significantly different.

The scavenging effect of DPPH radicals expressed as percentage versus concentration (mM) of extracts and standard (ascorbic acid, ASC) was plotted. The percentage of DPPH radical scavenging activity of all extracts was found to be dependent on isoflavone concentration. Using spectrophotometric analysis, the inhibition percentages of the KR and SM extracts were recorded as 94.14 ± 0.85% and 91.37 ± 0.27%, respectively, as shown in Table 5. Similarly, the KR extracts showed a higher inhibition percentage over 5 min, which explains why KR extracts showed higher inhibition than SM. Simultaneously, the equivalency of antioxidant activity was recorded in the order KR > SM using the EPR technique, as shown in Figure 4. The EPR spectra and percentage inhibition expressed the antioxidant activity of both extracts and their equivalency to ascorbic acid (Figure 4).

### 3.5. Total Polyphenol and Total Flavonoid Contents

The total polyphenol and flavonoid contents were calculated according to the equivalency of gallic acid (GA) and quercetin concentration, respectively. As shown in Table 5, the total polyphenol and total flavonoid contents were recorded in the order of KR extracts > SM extracts. The values shown in Table 5 indicate that TPC was 223.1 ± 19.07 and 330.5 ± 81.45 GA equivalent mmol/L in KR and SM extracts, respectively, whereas TFC was recorded as 201.2 ± 10.35 and 133.1 ± 11.3 quercetin equivalent (%) for KR and SM, respectively.

The antioxidant activity of phytoestrogens substantially contributes to the scavenging of free radicals released after oxidative stress. The potent antioxidant activities of the KR and SM extracts can be attributed to their isoflavone-rich content. Polyphenols and other flavonoids also play major roles in antitumor activity [63]. Moreover, phenolic compounds and isoflavones are known for their efficient radical scavenging activity, resulting from the hydroxyl groups at various positions and the ortho-dihydroxy structure in their B ring [64]. Thus, phenolic compounds significantly contribute to antioxidant activity [65,66]. As the concentration of isoflavone in KR extract was higher than that in SM, antioxidant activity was similarly determined to be higher in KR extracts. In summary, the KR and SM extracts showed high antioxidant activities, and these activities were in agreement with those of TFC determined in this study. However, this was not the case when the TPC of both extracts was analyzed. These results suggest that KR and SM extracts could also be used as sources of antioxidant and anticancer compounds. The extracts were further examined for their in vitro cytotoxic activity against three different cell lines.

### 3.6. Assessment of Cytotoxic Activities

**Figure 5:**
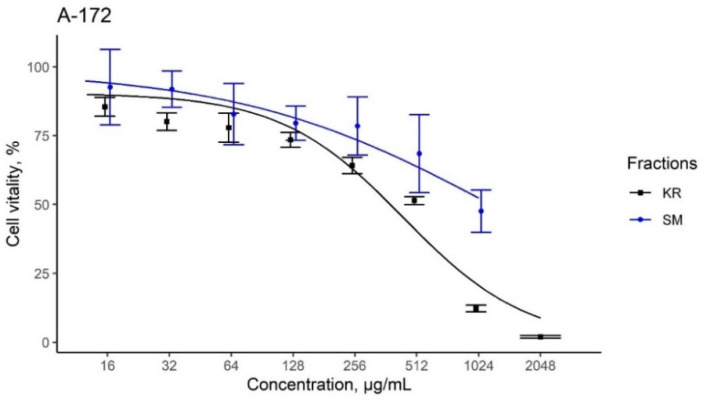

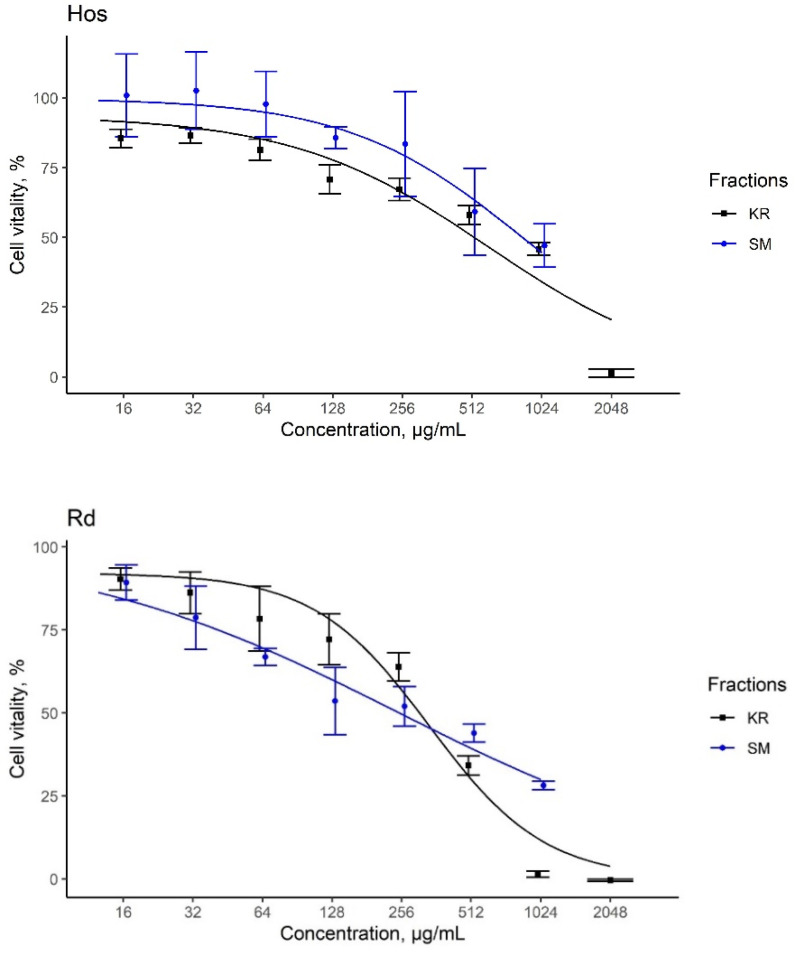
Evaluation of the effect of kudzu roots (KR) and soy molasses (SM) extracts on the viability of glioblastoma (A-172) cells, osteosarcoma (Hos) cells, and human embryonic rhabdomyosarcoma (Rd) cells. Data are shown as mean ± SD.

KR and SM extracts substantially decreased the viability of tumor cells, even at the lowest concentrations (−16–128 μg/mL), with a gradual increase observed in the effect when the concentrations reached 1–2 mg/mL. For instance, SM extracts exhibited a gradual increase in toxicity compared to the KR extracts. As shown in Figure 5, the effect of KR extract was similar in all three cell lines, with IC_50_ values of ~335–600 μg/mL. SM extract showed less pronounced toxic effects against glioblastoma and osteosarcoma cells (IC_50_ = 1213 and 848 μg/mL, respectively) (Table 6). However, the effect of SM extract on rhabdomyosarcoma cell lines was significantly more pronounced (IC_50_ = 244 μg/mL). This could be related to the specific sensitivity of Rd cells for the SM extract. However, this assumption requires further investigation.

Despite immense attempts to use chemotherapeutic or radiotherapeutic interventions, developing novel remedies to treat PSTs is still difficult. Owing to the increasingly complicated issue, the development of an alternative drug that also functions as a chemosensitizer and chemopreventive agent has garnered significant interest [9]. The in vitro cytotoxic activities of some phytoestrogenic sources and their effects on different cancer cell lines are shown in Table 7.

In the present study, in vitro biological analysis showed that KR and SM extracts decreased the proliferation and viability of glioblastoma, osteosarcoma, and embryonic RMS cell lines. As indicated by the MTT assay, the KR extracts were efficient in reducing the proliferation of all the aforementioned cell lines. The index of cytotoxicity (IC_50_), with a value of 337.4 μg/mL was lowest for Rd cell lines in the case of KR extracts. Similarly, KR extracts containing daidzein and genistein exhibited significant antiproliferative effects against cancer cell lines [69]. This can be attributed to the fact that these isoflavones may act as estrogen receptors (ERs). In the same context, the claim that cytotoxicity is correlated with the binding affinity of isoflavones to estrogenic receptors at different degrees based on their concentrations has been previously proven [28,71]. However, SM extracts had the lowest IC_50_ in Rd cell lines compared to that in A-172 and Hos cell lines. This could be associated with the higher content of both daidzein and genistein in the SM extracts.

A previous study performed by Nishio et al. demonstrated that a higher genistein concentration (10 µg/mL) could potentially decrease the cytotoxicity of natural killer cells [72]. Interestingly, the A-172 cell line seemed to be the least sensitive to isoflavones, compared to the Rd cell lines which were the most sensitive (Table 6). Hence, the mimicking of the mechanism of action of the human hormone estrogen by isoflavones would play a key role in their antiproliferative effect. Therefore, the selective mechanism of action of isoflavones individually or in combination may substantially contribute to inhibiting the growth of tumor cells, thereby suppressing the possibility of their metastatic activities.

Another study showed that daidzein inhibited the proliferation of GBM cells via CD44/moesin signaling [73]. Similarly, Pueraria tuberosa has demonstrated a marked anti-osteoporotic effect in ovariectomy-induced osteoporosis because of the antioxidant potential of its components, daidzein, and genistein [28,74]. Nonetheless, further investigation is required to analyze the effect of individual isoflavones on the viability of different cell lines and fill the gap in understanding their mechanisms of action.

## 4. Conclusions

KR and SM are potential phytochemical and food sources that demonstrate significant potential to be developed as pharmaceutical interventions against PSC cell lines, particularly against rhabdomyosarcoma tumor cells, in a dose-dependent manner. However, KR showed a significant antiproliferative effect against the growth of glioblastoma multiforme and osteosarcoma tumor cells compared with SM. HPLC-DAD-ESI-HRMS is a validated and sensitive method for the simultaneous quantification of a remarkable number of phytoestrogens and isoflavones in a short time. This approach successfully established an association between the identified isoflavones and in vitro physicochemical and cytotoxic parameters in the food and pharmaceutical industries. The total flavonoid content and antioxidant activities of the extracts were remarkably attributed to the isoflavone content. Overall, the presence of a high isoflavone content may act synergistically to demonstrate an antiproliferative effect and induce antioxidative stress effects against tumor cells, thereby preventing further metastatic complications. Future research is required for a better understanding of the mechanisms of action of the individual components of *Pueraria* species and soy products.

## Figures and Tables

**Table 1:** Regression equation and R^2^ of isoflavones.

Isoflavones	Regression Equation	R^2^
Puerarin	Y = 156.3464X+ 287792	0.99966
Daidzein	Y = 236.9975X + 9.2923	0.99995
Genistein	Y = 276.2041X + 19.7115	0.99979
Formonentin	Y = 202.7583X + 21.7709	0.99980
Biochanin A	Y = 256.2404X + 13.2775	0.99983

**Table 2:** Results of quantification of the isoflavones in ethyl acetate fractions of kudzu roots (KR) and soy molasses (SM).

Parameters	KR (%)	SM (%)
Puerarin	0.5 ± 0.002	NI
Daidzein	12 ± 0.004 ^a^	5.2 ± 0.005 ^b^
Genistein	2 ± 0.021 ^b^	5.7 ± 0.007 ^a^
Formonentin	0.2 ± 0.008	NI
Biochanin A	0.2 ± 0.001	NI
Sum	14.8 ± 0.078 ^a^	10.9 ± 0.006 ^b^

Data are shown as mean ± SD. NI: not identified. ^a,b^ Means that do not share the same letter in each column are significantly different.

**Table 3:** Major isoflavones identified in the extract of KR using HPLC-ESI-HRMS.

No.	Retention Time (*t*_R_, min)	Molecular Formula	Peak Area (EIC) % of Daidzein	Compound Name
1	6.1	C_21_H_20_O_9_	1.9	Puerarin *
2	7.1	C_21_H_20_O_10_	4.2	genistein-8-*C*-glucoside **
3	7.9	C_21_H_20_O_10_	1.7	genistein-7-*O*-glucoside **
4	9.6	C_15_H_10_O_5_	2.2	isomer of genistein **
5	10.4	C_15_H_10_O_4_	100	Daidzein *
6	10.6	C_16_H_12_O_5_	1.9	dihydroxy-methoxyisoflavone **
7	11.5	C_15_H_10_O_5_	15.8	Genistein *
8	11.6	C_16_H_12_O_6_	2.6	Tectorigenin **
9	12.5	C_17_H_14_O_5_	1.6	hydroxy-dimethoxyisoflavone **
10	12.9	C_16_H_12_O_4_	2.0	Formononetin *

* Identity determined based on MS (MS/MS) spectral and retention data using authentic standards. ** Tentative identification. Details are provided in the text. EIC: Extracted Ion Chromatogram.

**Table 4:** Major isoflavones identified in the extract of soy molasses (SM) using HPLC-ESI-HRMS.

No.	Retention Time (*t*_R_, min)	Molecular Formula	Peak Area (EIC) % of Daidzein	Compounds Name
1	7.9	C_21_H_20_O_10_	33.7	genistein-7-*O*-glucoside **
2	10.4	C_15_H_10_O_4_	100	Daidzein *
3	11.5	C_15_H_10_O_5_	114	Genistein *

* Identity determined based on MS (MS/MS) spectral and retention data using authentic standards. ** Tentative identification. Details are provided in the text. EIC: Extracted Ion Chromatogram.

**Table 5:** Evaluation of antioxidant activity, total polyphenol, and total flavonoid contents of kudzu roots (KR) and soy molasses (SM) extracts.

Parameters	KR	SM
DPPH Inhibition (%)	94.14 ± 0.85 ^a^	91.37 ± 0.27 ^b^
Ascorbic acid (mM equ.)	2.20 ± 0.05 ^a^	2.03 ± 0.02 ^b^
TPC GA equ. (mmol/L)	223.1 ± 19.07 ^b^	330.5 ± 81.45 ^a^
TFC Quercetin equ. (mmol/L)	201.2 ± 10.35 ^a^	133.1 ± 11.3 ^b^

Data are shown as mean ± SD. ^a,b^ Means that do not share the same letter in each column are significantly different.

**Table 6:** Index of cytotoxicity (IC_50_) of kudzu roots (KR) and soy molasses (SM) extracts.

	A-172-IC_50_ (μg/mL)	Hos-IC_50_ (μg/mL)	Rd-IC_50_ (μg/mL)
KR extracts	440.4	597.2	337.4
SM extracts	1212.9	847.6	244.4

**Table 7:** In vitro cytotoxic activities of some phytoestrogenic sources and their effect on different cancer cell lines.

Phytoestrogens/Bioactive Compounds	Concentrations/Cell Lines	Cytotoxic Effect	References
*Curcuma longa*/curcumin	Fibrosarcoma, liposarcoma, synovial sarcoma, and malignant fibrous histiocytoma/pleomorphic sarcoma/20 μM	↓ proliferation and viability of soft tissue sarcoma cells	[67]
*Pueraria lobata*/puerarin, daidzin, daidzein, and genistein	Gastric epithelial cell lines (GES-1)/10–100 μmol/L	↑ cell viability Protect GES-1 cells from injury induced by oxidative stress	[68]
*Pueraria mirifica* and *Pueraria lobata*/Eight isolated sub-fractions	breast, cervical, ovarian, colon, and liver cancer cell lines/0–5 μg/mL	Potential anti-proliferative effect on different cell lines No effect on normal human fibroblasts or Chang liver cells	[69]
*Pueraria lobata* (fermented vs. non-fermented)/7 different isoflavones	rat pheochromocytoma line 12/0–10 mg/mL	Protect against injury mediated by H_2_O_2_-induced oxidative stress	[29]
*Pueraria tuberosa*/genistein and daidzein	breast and ovarian cancer cell lines/31.5 to 500 μg/mL	In vitro cytotoxicity and anticancer activities	[28]
Formononetin	human osteosarcoma cell lines (U2OS)/0–80 μM	↓ proliferation of cancer cells activates apoptotic mechanisms against U2OS	[70]

↓ refers to decrease, ↑ refers to increase.

## Supplementary Materials

The following supporting information can be downloaded at: https://www.mdpi.com/article/10.3390/plants11060741/s1, Figure S1: CID spectrum of compound 6 (C_16_H_12_O_5_) ([M − H]^−^ = 283.0612, CE = 20 eV), Figure S2: CID spectrum of compound 6 (C_16_H_12_O_5_) ([M − H]^−^ = 283.0612, CE = 40 eV), Figure S3: CID spectrum of compound 8 (C_16_H_12_O_6_, tectorigenin) ([M + H]^+^ = 301.0707, CE = 40 eV), Figure S4: CID spectrum of compound 9 (C_17_H_14_O_5_) ([M − H]^−^ = 297.0768, CE = 20 eV), Figure S5: CID spectrum of compound 9 (C_17_H_14_O_5_) ([M − H]^−^ = 297.0768, CE = 40 eV), Figure S6: CID spectrum of compound 9 (C_17_H_14_O_5_) ([M + H]^+^ = 299.0914, CE = 40 eV), Figure S7: CID spectrum of compound 3 (C_21_H_20_O_10_, genistein-7-*O*-glucoside) ([M − H]^−^ = 431.0984, CE = 20 eV), Figure S8: CID spectrum of compound 3 (C_21_H_20_O_10_, genistein-7-*O*-glucoside) ([M − H]^−^ = 431.0984, CE = 40 eV), Figure S9: CID spectrum of compound 3 (C_21_H_20_O_10_, genistein-7-*O*-glucoside) ([M + H]^+^ = 433.1129, CE = 50 eV), Figure S10: Fragment of CID spectrum of compound 3 (C_21_H_20_O_10_, genistein-7-*O*-glucoside) ([M + H]^+^ = 433.1129, CE = 50 eV), Figure S11: CID spectrum of compound 3 (C_21_H_20_O_10_, genistein-7-*O*-glucoside) ([M + H]^+^ = 433.1129, CE = 60 eV), Figure S12: CID spectrum of compound 2 (C_21_H_20_O_10_, genistein-8-*C*-glucoside) ([M − H]^−^ = 431.0984, CE = 20 eV), Figure S13: CID spectrum of compound 2 (C_21_H_20_O_10_, genistein-8-*C*-glucoside) ([M − H]^−^ = 431.0984, CE = 40 eV), Figure S14: CID spectrum of compound 2 (C_21_H_20_O_10_, genistein-8-*C*-glucoside) ([M + H]^+^ = 433.1129, CE = 20 eV), Figure S15: CID spectrum of compound 2 (C_21_H_20_O_10_, genistein-8-*C*-glucoside) ([M + H]^+^ = 433.1129, CE = 30 eV), Figure S16: CID spectrum of compound 2 (C_21_H_20_O_10_, genistein-8-*C*-glucoside) ([M + H]^+^ = 433.1129, CE = 40 eV), Figure S17: CID spectrum of compound 2 (C_21_H_20_O_10_, genistein-8-*C*-glucoside) ([M + H]^+^ = 433.1129, CE = 50 eV), Figure S18: CID spectrum of compound 2 (C_21_H_20_O_10_, genistein-8-*C*-glucoside) ([M + H]^+^ = 433.1129, CE = 60 eV).
