# Supplementary material for: Antioxidant and Cytotoxic Activities of Kudzu Roots and Soy Molasses against Pediatric Tumors and Phytochemical Analysis of Isoflavones Using HPLC-DAD-ESI-HRMS
Source: Plants (Basel). 2022 Mar 10;11(6):741. doi: 10.3390/plants11060741 (PMC8955742; doi:10.3390/plants11060741)
Supplement: Supplementary file 1 [file plants-11-00741-s001.zip › plants-1624724-supplementary.pdf]

plants

Article

Antioxidant and Cytotoxic Activities of Kudzu Roots and Soy Molasses against Pediatric Tumors and Phytochemical Analysis of Isoflavones Using HPLC-DAD-ESI-HRMS

Saied A. Aboushanab [1,2,*], Vadim A. Shevyrin [1,2], Grigory P. Slesarev [1,2], Vsevolod V. Melekhin [2,3,4], Anna V. Shcheglova [2,3], Oleg G. Makeev [3,4], Elena G. Kovaleva [1,2] and Ki Hyun Kim [5,*]

1 Institute of Chemical Engineering, Ural Federal University Named after the First President of Russia B. N. Yeltsin, Mira 19, 620002 Yekaterinburg, Russia; vadim.shevyrin@gmail.com (V.A.S.); grixa2000@yandex.ru (G.P.S.); e.g.kovaleva@urfu.ru (E.G.K.)
2 Innovative Center of Chemical and Pharmaceutical Technologies, Laboratory of Organic Synthesis, Ural Federal University Named after the First President of Russia B. N. Yeltsin, Mira 19, 620002 Yekaterinburg, Russia; melekhinvv@mail.ru (V.V.M.); ann257goldfinch@gmail.com (A.V.S.)
3 Department of Biology, Ural State Medical University, Repina 3, 620014 Yekaterinburg, Russia; larim@mail.ru
4 Department of Gene and Cell Therapy, Institute for Medical Cell Technologies, Karla Marksa 22a, 620026 Yekaterinburg, Russia
5 School of Pharmacy, Sungkyunkwan University, Suwon 16419, Korea
* Correspondence: sabushanab@urfu.ru (S.A.A.); khkim83@skku.edu (K.H.K.); Tel.: +82-31-290-7700 (K.H.K.)

Supporting Information

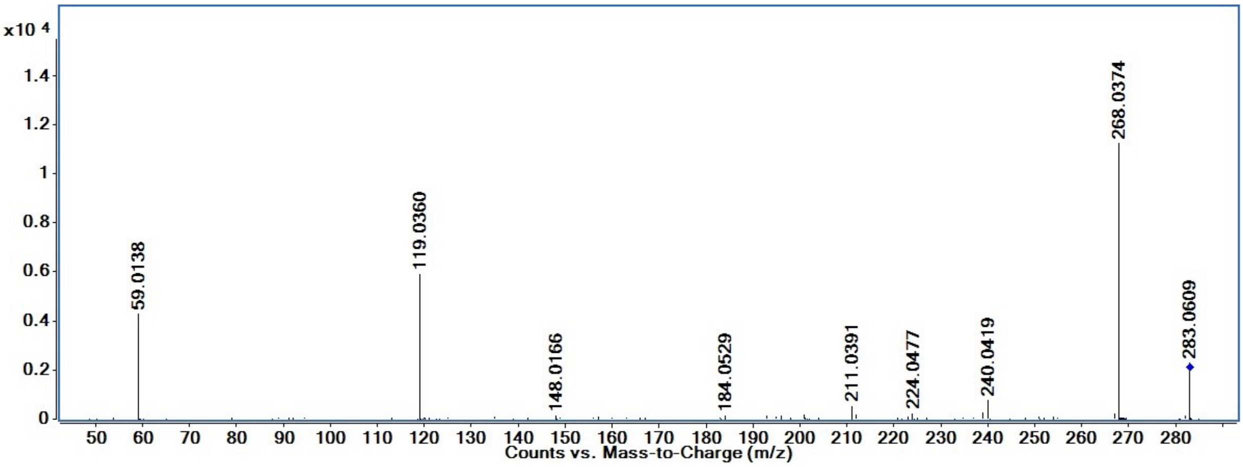

Figure S1. CID spectrum of compound 6 ($C_{16}H_{12}O_5$) ($[M\text{-}H]^-$ = 283.0612, CE = 20 eV)

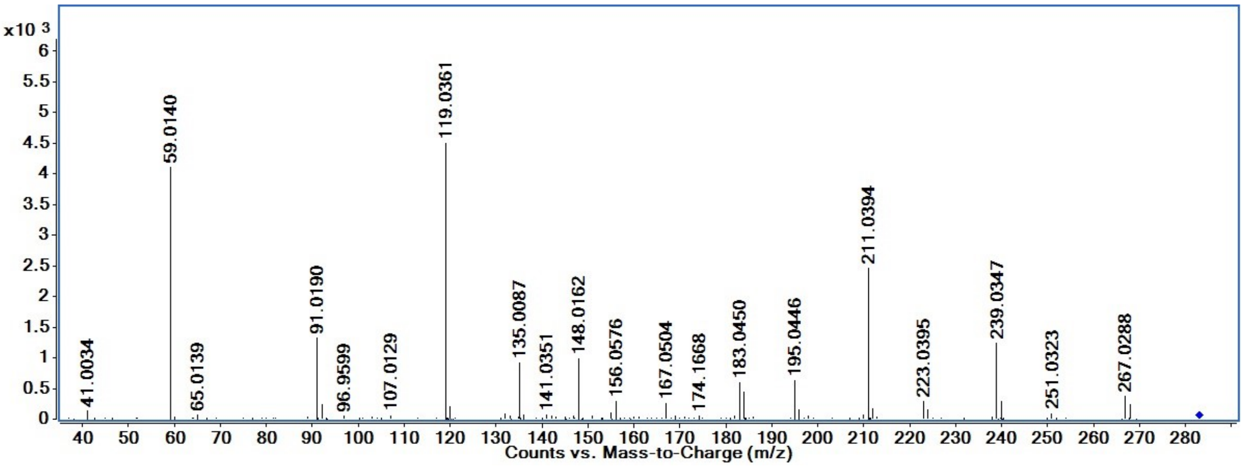

Figure S2. CID spectrum of compound 6 ($C_{16}H_{12}O_5$) ($[M\text{-}H]^-$ = 283.0612, CE = 40 eV)

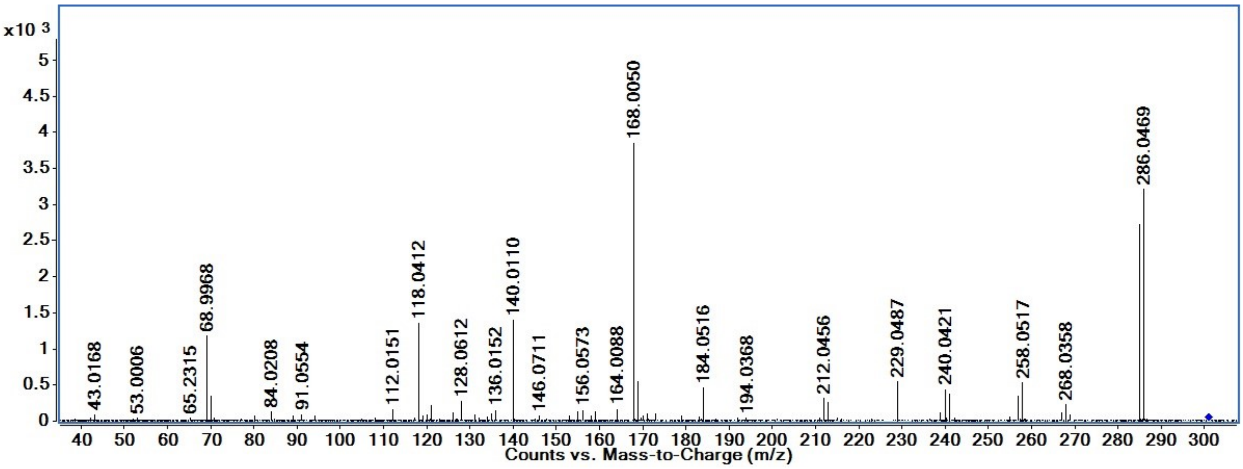

Figure S3. CID spectrum of compound 8 ($C_{16}H_{12}O_6$, tectorigenin) ($[M+H]^+$ = 301.0707, CE = 40 eV)

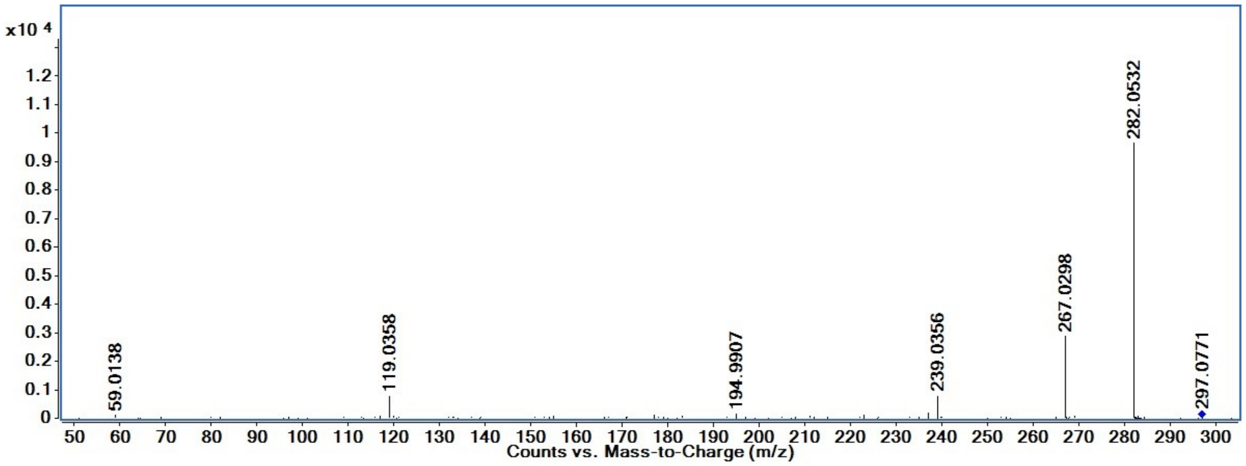

Figure S4. CID spectrum of compound 9 ($C_{17}H_{14}O_5$) ($[M-H]^-$ = 297.0768, CE = 20 eV)

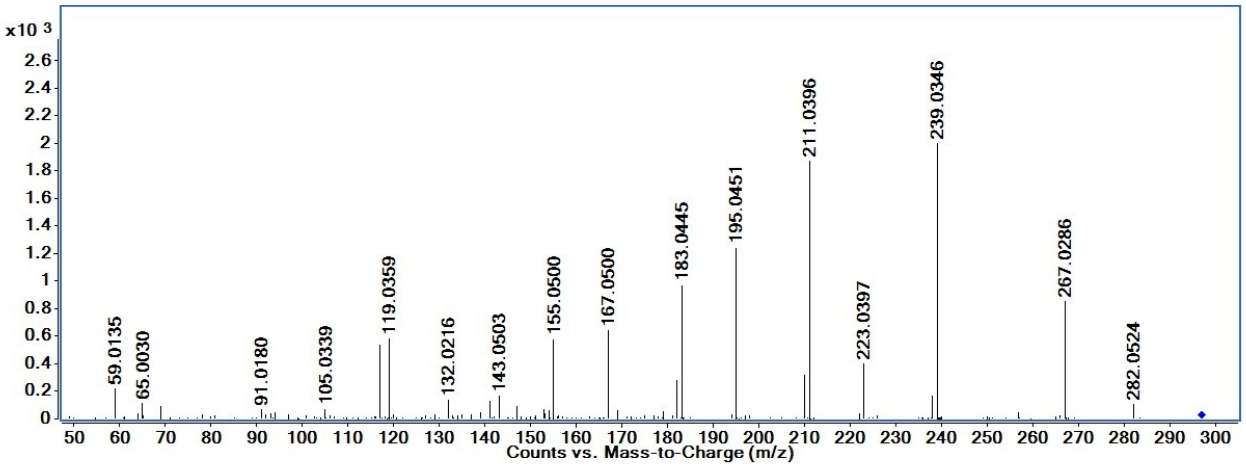

Figure S5. CID spectrum of compound 9 ($C_{17}H_{14}O_5$) ($[M-H]^-$ = 297.0768, CE = 40 eV)

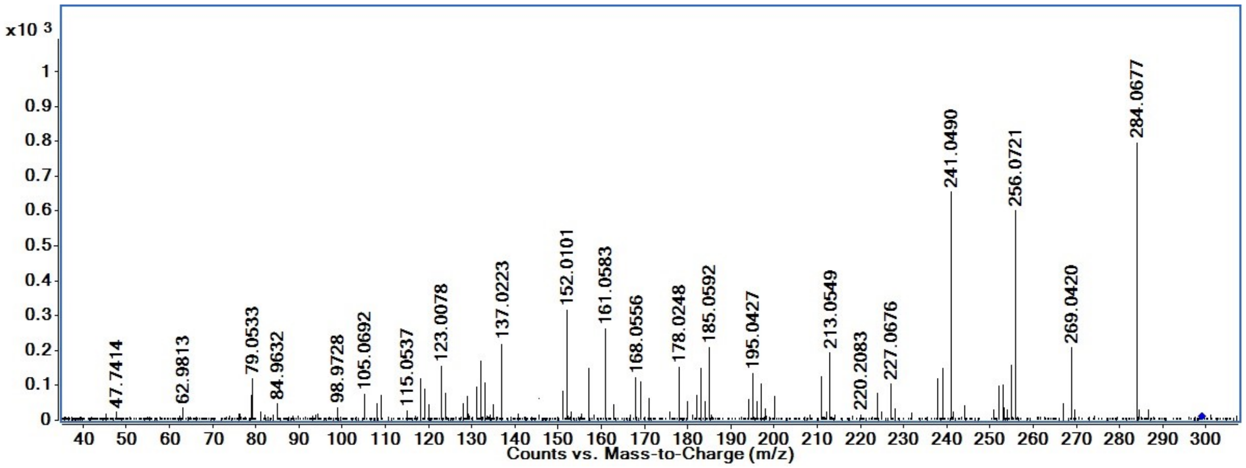

Figure S6. CID spectrum of compound 9 ($C_{17}H_{14}O_5$) ($[M+H]^+$ = 299.0914, CE = 40 eV)

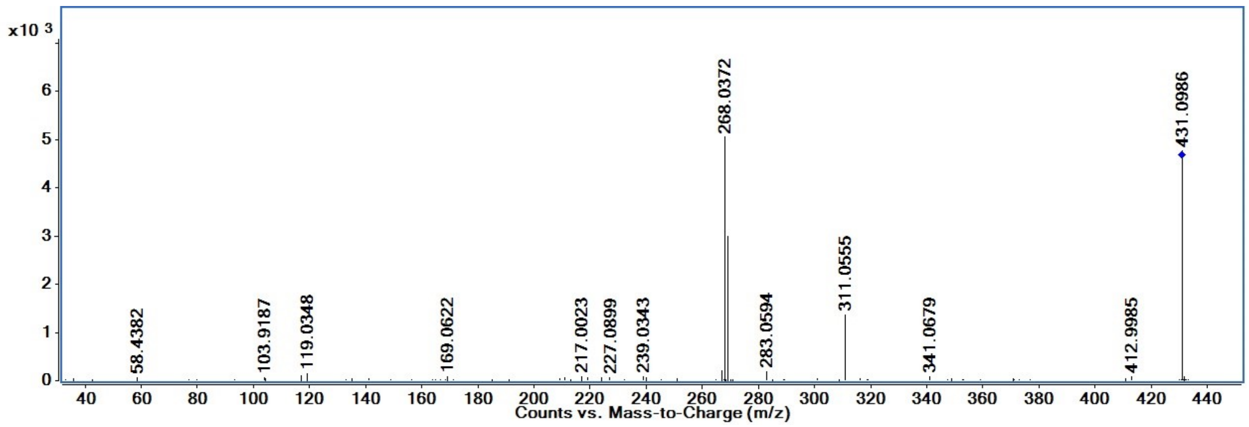

Figure S7. CID spectrum of compound 3 ($C_{21}H_{20}O_{10}$, genistein-7-*O*-glucoside) ($[M-H]^-$ = 431.0984, CE = 20 eV)

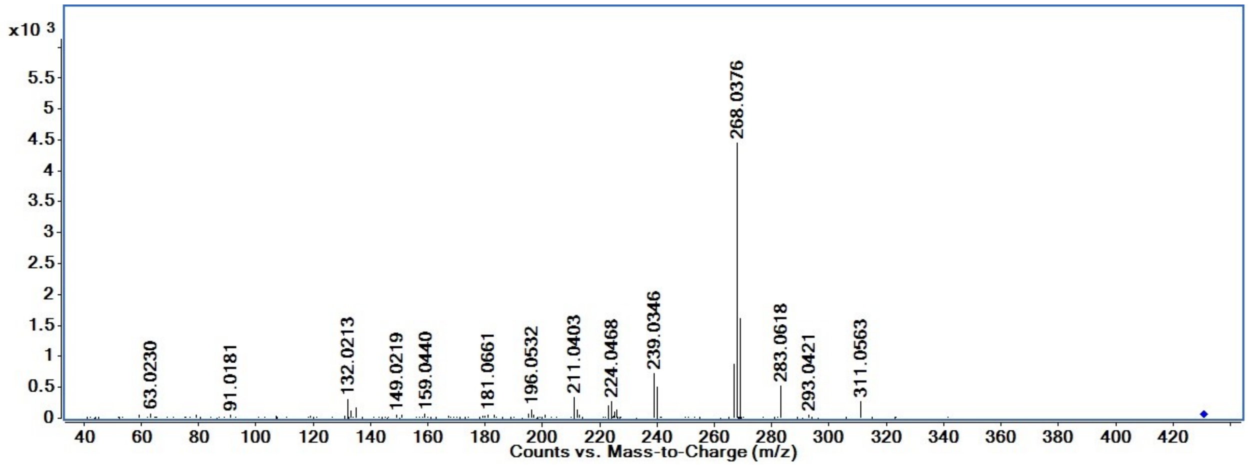

Figure S8. CID spectrum of compound 3 ($C_{21}H_{20}O_{10}$, genistein-7-*O*-glucoside) ($[M-H]^-$ = 431.0984, CE = 40 eV)

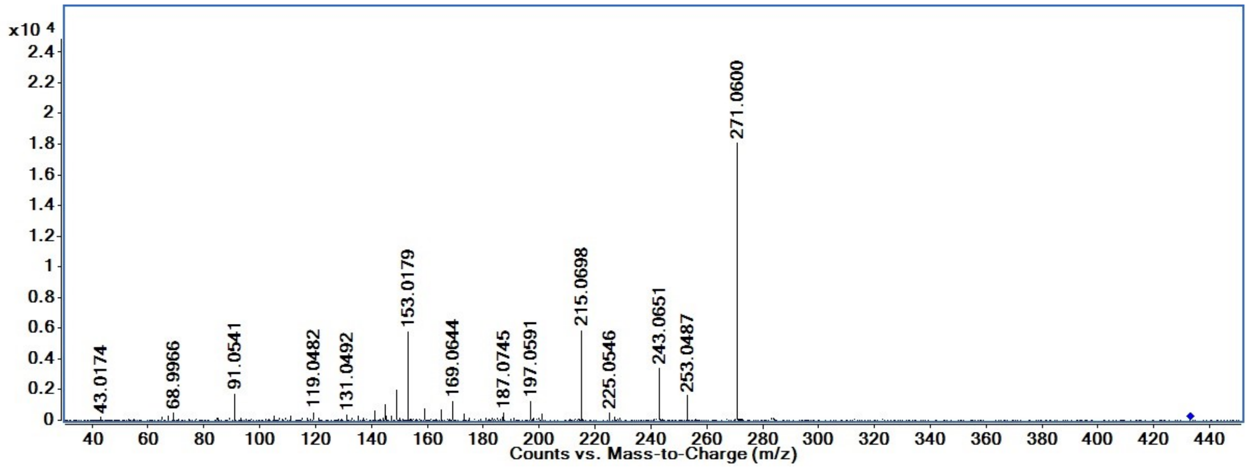

Figure S9. CID spectrum of compound 3 ($C_{21}H_{20}O_{10}$, genistein-7-*O*-glucoside) ($[M+H]^+$ = 433.1129, CE = 50 eV)

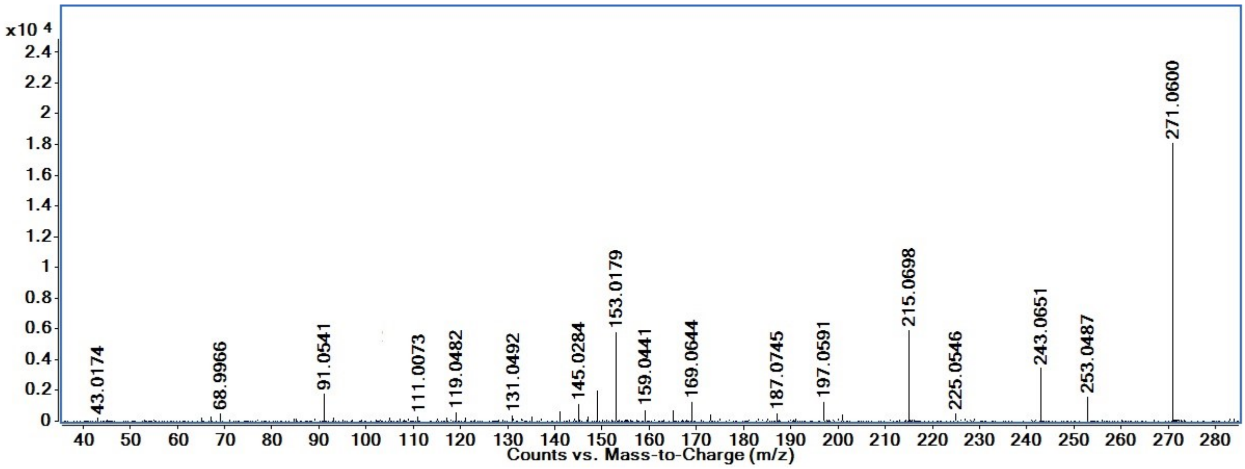

Figure S10. Fragment of CID spectrum of compound 3 ($C_{21}H_{20}O_{10}$, genistein-7-*O*-glucoside) ($[M+H]^+$ = 433.1129, CE = 50 eV)

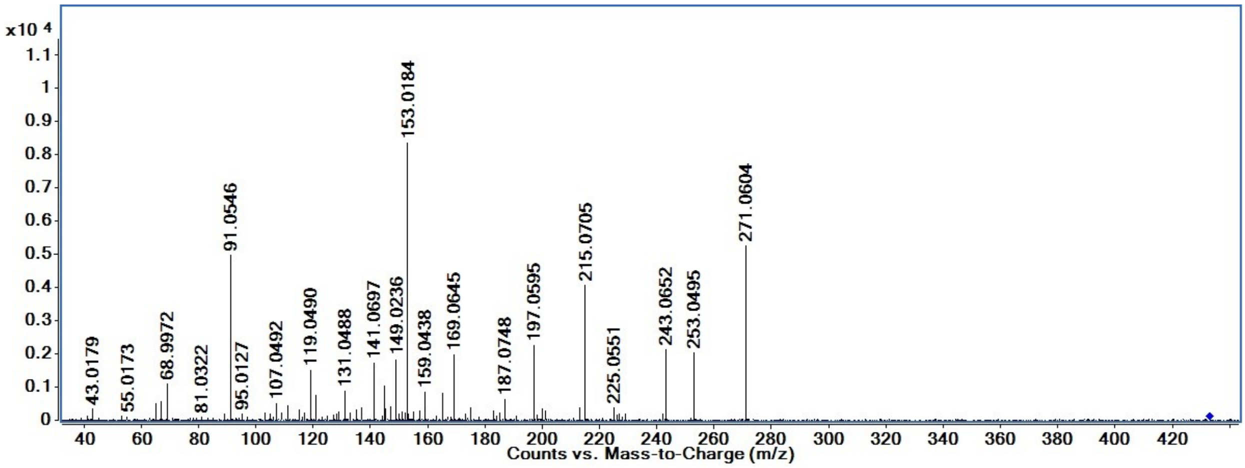

Figure S11. CID spectrum of compound 3 ($C_{21}H_{20}O_{10}$, genistein-7-*O*-glucoside) ($[M+H]^+$ = 433.1129, CE = 60 eV)

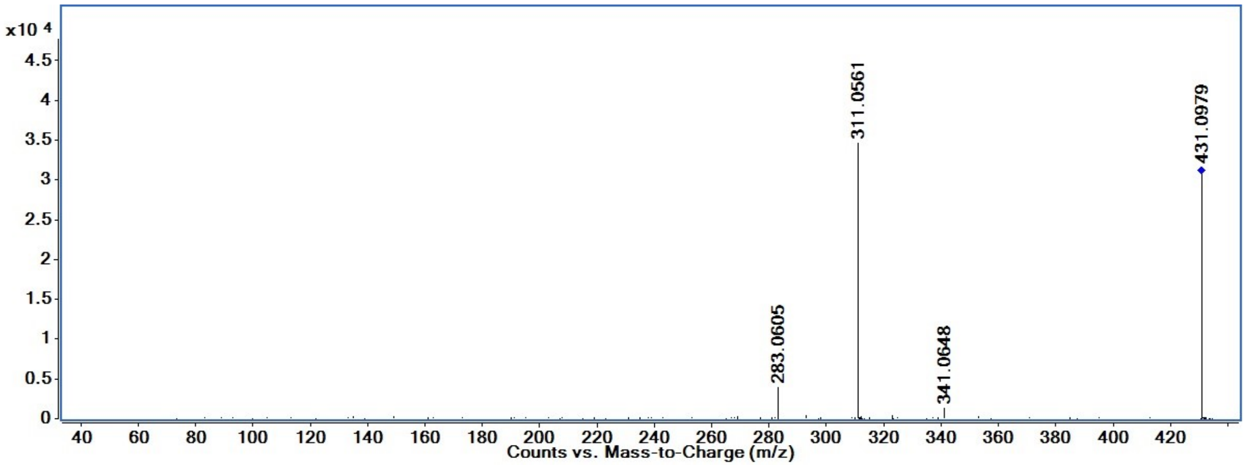

Figure S12. CID spectrum of compound 2 ($C_{21}H_{20}O_{10}$, genistein-8-*C*-glucoside) ($[M-H]^-$ = 431.0984, CE = 20 eV)

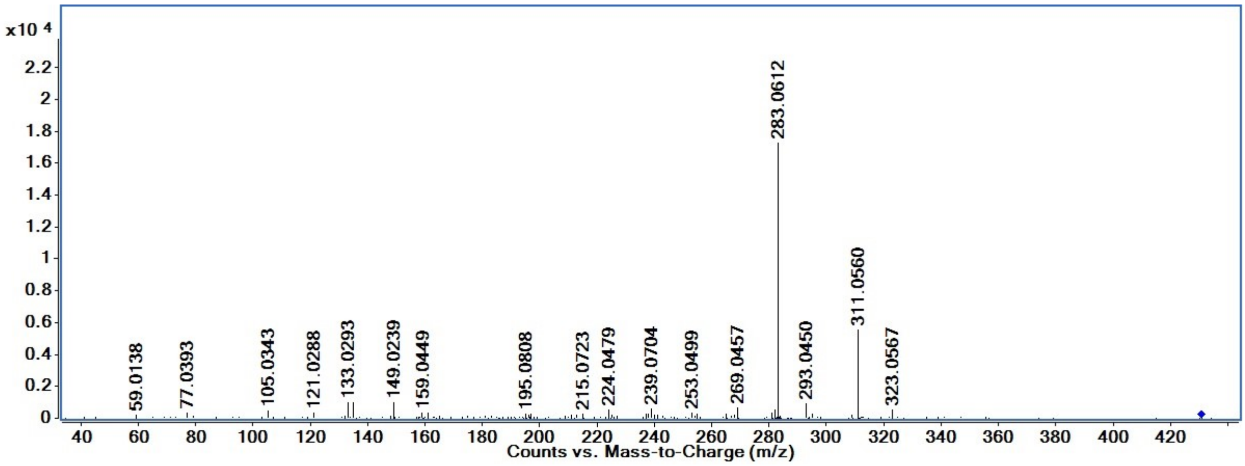

Figure S13. CID spectrum of compound 2 ($C_{21}H_{20}O_{10}$, genistein-8-*C*-glucoside) ([M-H]$^-$ = 431.0984, CE = 40 eV)

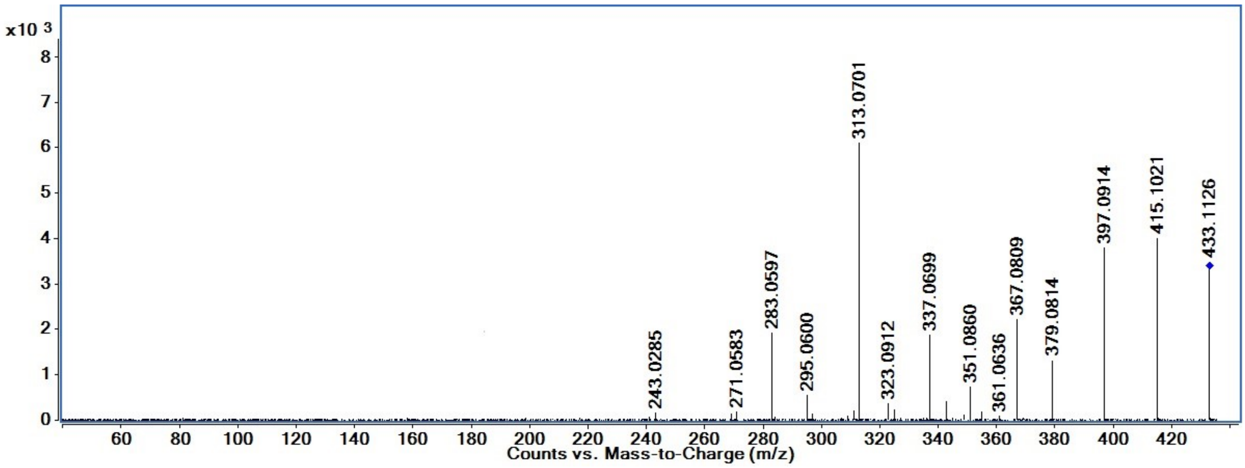

Figure S14. CID spectrum of compound 2 ($C_{21}H_{20}O_{10}$, genistein-8-*C*-glucoside) ([M+H]$^+$ = 433.1129, CE = 20 eV)

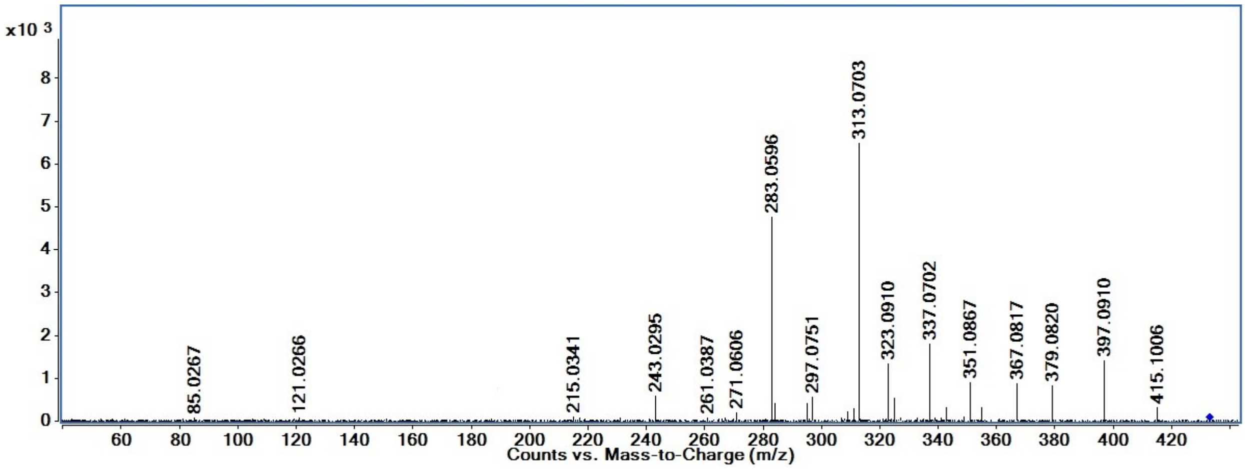

Figure S15. CID spectrum of compound 2 ($C_{21}H_{20}O_{10}$, genistein-8-*C*-glucoside) ([M+H]$^+$ = 433.1129, CE = 30 eV)

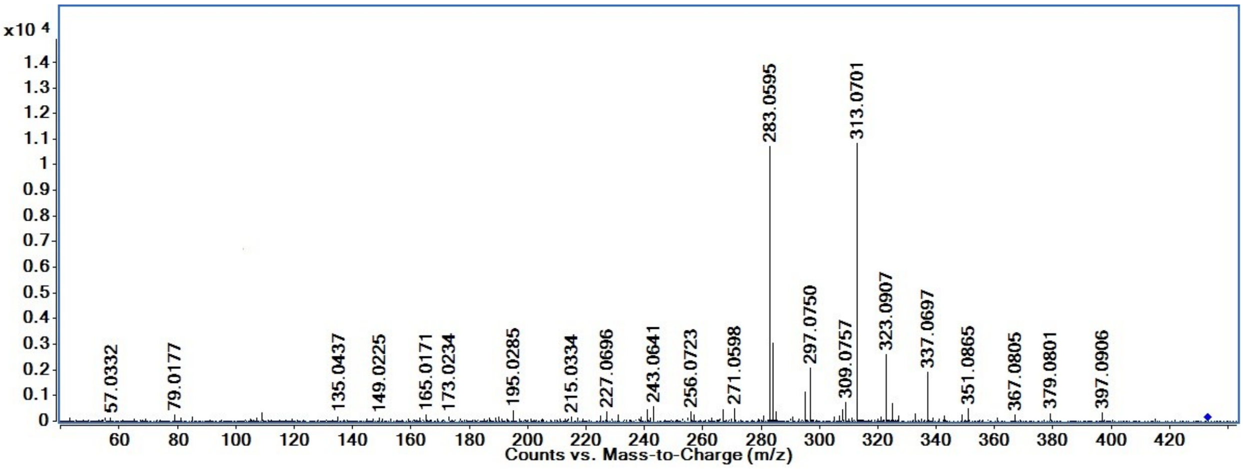

Figure S16. CID spectrum of compound 2 ($C_{21}H_{20}O_{10}$, genistein-8-*C*-glucoside) ($[M+H]^+$ = 433.1129, CE = 40 eV)

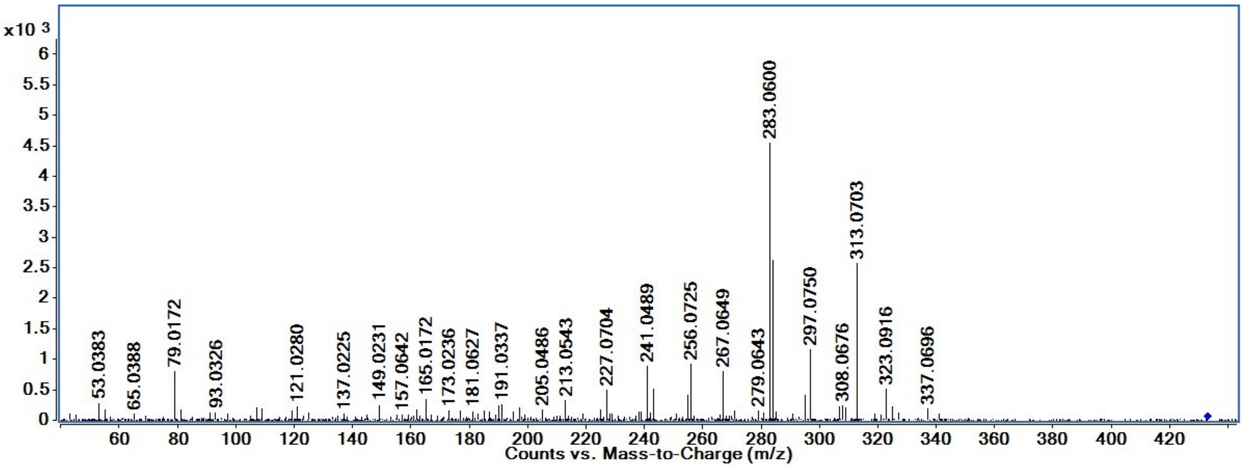

Figure S17. CID spectrum of compound 2 ($C_{21}H_{20}O_{10}$, genistein-8-*C*-glucoside) ($[M+H]^+$ = 433.1129, CE = 50 eV)

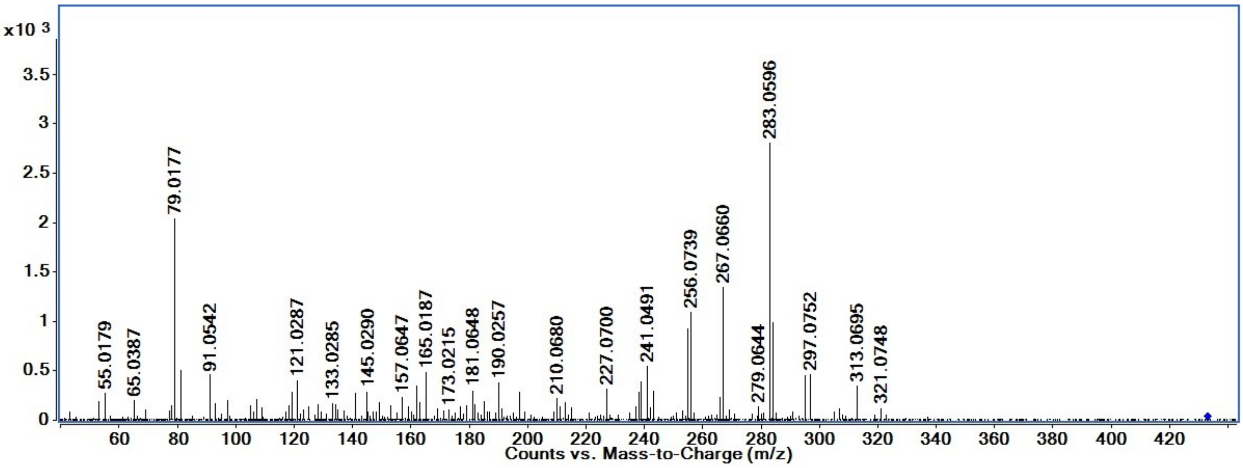

Figure S18. CID spectrum of compound 2 ($C_{21}H_{20}O_{10}$, genistein-8-*C*-glucoside) ($[M+H]^+$ = 433.1129, CE = 60 eV)
